# Inhibition of RhoA/Rho kinase signaling pathway by fasudil protects against kainic acid‐induced neurite injury

**DOI:** 10.1002/brb3.2266

**Published:** 2021-06-22

**Authors:** Yingchun Xiang, Yumiao Niu, Yacong Xie, Shishuo Chen, Feng Zhu, Weida Shen, Ling‐Hui Zeng

**Affiliations:** ^1^ Department of Pharmacy Zhejiang Hospital Hangzhou Zhejiang China; ^2^ Department of Pharmacology School of Medicine Zhejiang University City College Hangzhou Zhejiang China

**Keywords:** fasudil, kainic acid, neurite outgrowth, RhoA/Rho kinase pathway, seizure

## Abstract

**Aim:**

RhoA/Rho kinase pathway is essential for regulating cytoskeletal structure. Although its effect on normal neurite outgrowth has been demonstrated, the role of this pathway in seizure‐induced neurite injury has not been revealed. The research examined the phosphorylation level of RhoA/Rho kinase signaling pathway and to clarify the effect of fasudil on RhoA/Rho kinase signaling pathway and neurite outgrowth in kainic acid (KA)‐treated Neuro‐2A cells and hippocampal neurons.

**Method:**

Western blotting analysis was used to investigate the expression of key proteins of RhoA/Rho kinase signaling pathway and the depolymerization of actin. After incubated without serum to induce neurite outgrowth, Neuro‐2A cells were fixed, and immunofluorescent assay of rhodamine‐phalloidin was applied to detect the cellular morphology and neurite length. The influence of KA on neurons was detected in primary hippocampal neurons. Whole‐cell patch clamp was conducted in cultured neurons or hippocampal slices to record action potentials.

**Result:**

KA at the dose of 100–200 μmol/L induced the increase in phosphorylation of Rho‐associated coiled‐coil‐containing protein kinase and decrease in phosphorylation of Lin11, Isl‐1 and Mec‐3 kinase and cofilin. The effect of 200 μmol/L KA was peaked at 1–2 hours, and then gradually returned to baseline after 8 hours. Pretreatment with Rho kinase inhibitor fasudil reversed KA‐induced activation of RhoA/Rho kinase pathway and increase in phosphorylation of slingshot and 14‐3‐3, which consequently reduced the ratio of G/F‐actin. KA treatment induced inhibition of neurite outgrowth and decrease in spines both in Neuro‐2a cells and in cultured hippocampal neurons, and pretreatment with fasudil alleviated KA‐induced neurite outgrowth inhibition and spine loss.

**Conclusion:**

These data indicate that inhibiting RhoA/Rho kinase pathway might be a potential treatment for seizure‐induced injury.

## Graphical abstract

Kainic acid at the dose of 100 to 200 μmol/L induced the increase in phosphorylation of ROCK and decrease in phosphorylation of LIMK and cofilin. Pretreatment with Rho kinase inhibitor Fasudil reversed KA‐induced activation of RhoA/Rho kinase pathway and increase in phosphorylation of SSH and 14‐3‐3. Pretreatment with Fasudil alleviated KA‐induced neurite outgrowth inhibition and spine loss.

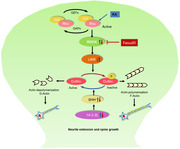

## 1 INTRODUCTION

Epilepsy is a group of neurological disorders characterized by transient disturbance caused by abnormal excessive or synchronous neuronal discharges (Fisher et al., 2005). Recurrent epileptic seizures lead to a series of neurobiological, cognitive, psychological and social consequences. Cognitive impairment is one of the commonest comorbidities of epilepsy. Though recent treatments are effective in controlling seizures, their impacts on cognitive function remain unsatisfactory (Dias et al., 2017). Apart from the effects of chronic epilepsy on brain anatomy and physiology, the underlying brain dysfunction responsible for epilepsy also contributes significantly to cognitive morbidity in a dependent way. An 8‐ to 9‐year follow‐up of adolescents with diagnosed epilepsy in childhood showed that up to 39% of them also suffered from neurodevelopmental disorders (Baca et al., 2011). Cognitive impairments shown in epileptic children include difficulties in learning, memory, problem solving, as well as concept formation. Anxiety, depression and attention deficit disorders are the most common psychiatric comorbidities (Gulati et al., 2014). Thus, cognitive impairment and psychiatric comorbidities have pronounced impacts on long‐term life quality of people with childhood‐onset epilepsy.

It has been widely considered that plasticity of dendritic spines is one of the key mechanisms and substrates of cognitive function (McCann & David, 2017; Park et al., 2017 ). It has been reported that kainic acid (KA)‐induced seizures caused irreversible dendritic beading morphological change and loss of spines (Zeng et al., 2007). Furthermore, KA‐induced seizures also resulted in activation of actin‐depolymerizing factors and a corresponding decrease in filamentous actin (Zeng et al., 2007). The actin cytoskeleton is crucial for neurite growth cone, spine development, and formation of synapses in neurons (Eiseler et al., 2010; Yamada et al., 2013 ). Cofilin is one of the key proteins of the RhoA/Rho kinase pathway which is essential for regulating cytoskeletal structure (Patel et al., 2017). At present, pharmacological inhibitors of Rho‐associated protein kinase 2 (ROCK2) have been extensively investigated. Among them, fasudil is the best characterized and most frequently used (Feng et al., 2016). Previous studies have revealed that fasudil has antiepileptic effects and significantly reduces the duration of epileptic seizure as well as recovery latency for righting reflex, and also prolongs the onset of seizures in acute seizure models (Inan & Büyükafsar, 2008; Xu et al., 2014 ). These results indicated that RhoA/Rho kinase pathway might play an important role in regulating dendritic structure after seizures and that inhibitors of RhoA/Rho kinase pathway have potential clinical application for preventing or reversing seizure‐induced cognitive impairment and psychiatric comorbidities.

Neuro‐2a is a fast‐growing mouse neuroblastoma cell line, with the potential to differentiate into neuron‐like cells and easy to culture. In the present study, the effect of RhoA/Rho kinase signaling pathway on neurite morphology in KA‐treated Neuro‐2a cells was investigated. Moreover, the impact of RhoA/Rho kinase signaling pathway on neurite morphology in the KA‐treated primary cultured hippocampal neurons was also examined. The study aimed at illustrating the effects of the specific intracellular signaling and mechanistic elements on seizure‐induced dendrite injury and laying the foundation for a potential treatment of seizure‐induced cognitive impairment.

## 2 MATERIALS AND METHODS

### 2.1 Animals

Pregnant Sprague‐Dawley rats (SLRC laboratory animal) were used for the experiments. Rats were housed under a 12/12 hours light/dark cycle at 25°C with free access to food and water. All experiments followed the Guidelines for the Care and Use of Laboratory Animals of Zhejiang University and were approved by the Ethics Committee of the Zhejiang University.

### 2.2 Cells and drug treatment

Neuro‐2a cell line was purchased from the Type Culture Collection of the Chinese Academy of Sciences (Shanghai, China). It was cultured in 45% DMEM (high glucose) (Gibco, Gaithersburg, MD, USA) and 45% opti‐MEM supplemented with heat‐inactivated 7% fetal bovine serum (FBS, Invitrogen, USA), 100 U/mL penicillin and 100 U/mL streptomycin (Genom, Hangzhou, China), at 37°C with 5% CO_2_ and 95% O_2_.

KA and Fasudil hydrochloride (HA‐1077) were obtained from Nanocs Inc. (New York, USA) and Selleck (Texas, USA), respectively. They were both initially dissolved in PBS, stored at −4°C, and diluted by medium immediately before treatment, as described previously (Zhu et al., 2011).

### 2.3 Primary culture of hippocampal neurons

The cultured hippocampal neurons were obtained from the 18‐day embryonic (E18) Sprague–Dawley rats. Glass coverslips (20 mm in diameter) were placed in dishes and coated with 0.5 mg/mL poly‐L‐lysine hydrobromide (Sigma‐Aldrich, Saint Louis, MO, USA) for 2 hours and then washed with phosphate‐buffered saline (PBS). Briefly, the pregnant rats were anesthetized with 10% chloral hydrate. Embryos were then transferred to a 10 cm dish with cold Hank's balanced salt solution (HBSS, no calcium, no magnesium, no phenol red) (Gibco, 14175079, USA). The brains of embryos were removed to a new dish with cold HBSS. Hippocampi were dissected and mechanically dissociated, and the tissues were then treated with 2 μg/mL papain solution (Sangon Biotech, Shanghai, China) to isolate hippocampal neurons. The hippocampal neuron cells were seeded at a density of 1 × 10^5^ cells per cm^2^ onto the prearranged dishes with plating medium, which consisted of DMEM/F12 (Gibco, 11330032, USA), 10% horse serum (Gibco™ Heat‐Inactivated, New Zealand Origin), and 1% penicillin–streptomycin (Gibco™, 15070063, USA). Neurons were maintained at 37°C in an incubator containing humidified air with 5% CO_2_ and 95% O_2_. After 5 hours, the plating medium was placed with neurobasal medium, which was composed of neurobasal medium (Gibco™, 21103049, USA), 2% B27 (Gibco™, 17504044, USA), 1% penicillin—streptomycin, and 1% GlutaMAX 100× (Gibco™, 35050061, USA). Cytarabine (Ara‐C, Pfizer, USA) was added to the culture medium at a final concentration of 2.5 μg/mL, and all of the culture medium was replaced after 2 days. Then medium was renewed every 4 days in the following 2 weeks.

### 2.4 Active RhoA detection

The detection of active RhoA was conducted according to the protocol of Active Rho Detection Kit purchased from Cell Signaling Technology. Briefly, the hippocampus tissue was isolated and lysated with 500 μL 1× Lysis Buffer(add 1 mM PMSF before using). BCA method was used to measure the concentration of protein. First, the Glutathione Resin suspension was centrifuged to discard the liquid, then 400 μg GST‐Rhotekin‐RBD was added before the sample was added to the spin cup. After shaked at 4°C for 1 hour, sample was washed with the Lysis Buffer twice and transferred to a new collecting cup, 50 μL 2× reducing sample buffer (200 mM DTT before use) was added to the spin cup and vortexed, incubated at room temperature for 2 minutes, centrifuged at 4°C. Finally, the liquid was collected, boiled for 5 minutes, and analyzed by western blot.

### 2.5 Western blot analysis

The differentiation of cells was induced by serum deprivation for 1 hour for western blot analysis of key proteins in the RhoA/Rho kinase pathway. Then total protein was extracted from cells at various time points and dosages after saline or KA treatment. In experiments of investigating the effects of Rho kinase inhibitors on RhoA/Rho kinase pathway, fasudil (200 μmol/L) was applied 2 hours before KA treatment. The cells were sonicated individually in RIPA lysis buffer supplemented with 1％ protease and 1％ phosphatase inhibitors. Protein concentration was determined with a BCA Protein Assay Kit (Beyotime, Shanghai, China). Fifty micrograms of proteins were separated by 12% SDS‐PAGE and transferred to PVDF membranes. After incubation with the rabbit anti‐ROCK2(PhosphoT249) (1:1000; Abcam, Cambridge, MA, UK), anti‐phospho‐LIMK1 (Thr508)/LIMK2 (Thr505), anti‐phospho‐cofilin (Ser3), anti‐slingshot‐1L (Ser‐978), and anti‐phospho‐(Ser)14‐3‐3 binding motif antibody (1:1000; Cell Signaling Technology, Beverly, MA, USA), the membranes were labeled with horseradish peroxidase secondary antibody and visualized with ECL reagent (Pierce, Rockford, IL, USA). The membranes were then re‐probed and incubated with the rabbit anti‐ROCK2, anti‐LIMK1, anti‐cofilin, anti‐SSH1(E1K3W), and anti‐14‐3‐3)pan(antibody (1:1000; Cell Signaling Technology, Beverly, MA, USA). Intensity of each lane in each blot was measured quantitatively by Image J. At least three independent trials were conducted for each experiment.

Analysis of the ratio of F‐actin to G‐actin was also made by western blot according to a previously published method (Zeng et al., 2007). After serum deprivation for 1 hour and then drug treatment (refer to the previous paragraph for drug delivery), cells were collected and homogenized in G‐actin lysis buffer and then centrifuged at 15,000 rpm for 30 minutes. The supernatant was used to determine the amount of G‐actin. The pellets were resuspended in G‐actin lysis buffer plus an equal volume of F‐actin, and gently mixed every 5 minutes for 1 hour to depolymerize F‐actin. The samples were centrifuged at 15,000 rpm for 30 minutes, and the supernatant was also used for measurement of F‐actin. G‐actin and F‐actin samples were both determined and analyzed according to western blot analysis. The protein samples were always kept on ice during experiments. The formulas of G‐actin and F‐actin lysis buffer were as follows: G‐actin lysis buffer: 10 mmol/L K_2_HPO_4_, 100 mmol/L NaF, 50 mmol/L KCl, 2 mmol/L MgCl_2_, 1 mmol/L EGTA, 0.2 mmol/L dithiothreitol, 0.5% Triton X‐100, 1 mol/L sucrose, pH 7.0; F‐actin lysis buffer: 1.5 mol/L guanidine hydrochloride, 1 mol/L sodium acetate, 1 mmol/L CaCl_2_, 1 mmol/L ATP, and 20 mmol/L Tris‐HCl, pH7.5.

### 2.6 Measuring of the neurite outgrowth in Neuro‐2a cells under different interface contrast microscopy

The Neuro‐2a cells were seeded onto glass coverslips and exposed to serum deprivation‐induced differentiation for 1 hour. Then the cells were divided into three groups and were treated with PBS, 200 μmol/L KA, and 200 μmol/L KA with pretreatment of 200 μmol/L fasudil for 2 hours. The cells were observed under a Nikon Eclipse Ts2R microscope (Nikon, Japan) at different time points of 1 and 4 hours after KA treatment. The images were acquired by NIS‐Elements (Nikon, Japan) and analyzed with Image J.

### 2.7 Rhodamine‐phalloidin immunofluorescence

At predetermined time points, Neuro‐2a cells and primary hippocampal neurons were fixed at room temperature for 10−30 minutes in 4% formaldehyde (in PFA) After several washes with PBS, 0.1% triton X‐100 (in PBS) was added into the fixed cells for 3−5 minutes. The cells were blocked by incubation at room temperature for 1−2 hours in 1% bull serum albumin in PBS. The cells were then washed with PBS for 3 times followed by incubation overnight at 4°C with rhodamine‐phalloidin (1:1000; Abcam, Cambridge, MA, UK). The cells were then washed with PBS to remove the unbound reagent and were then mounted in Vectashield. The cells were visualized with a confocal laser scanning microscope (Olympus, Japan).

### 2.8 Calculation of neurite length and spine number

Images either taken by optical or confocal microscope for each group were analyzed by Image J. Images with clear background and uniform cell density were selected with a precondition that the neurites were not connected with other cells. The length of the longest neurites of each cell was calculated using NeuronJ function of Image J. One hundred cells were observed and measured in each group. The numbers of the spines in each cell neurites were also counted.

### 2.9 Electrophysiology

Thirty‐ to sixty‐day‐old Sprague–Dawley rats were decapitated under anesthesia with isoflurane. Brains were removed and transferred into a prechilled protective solution that contained (in mM) 235 sucrose, 2.5 KCl, 1.25 NaH_2_PO_4_, 0.5 CaCl_2_, 7 MgCl_2_, 20 glucose, 26 NaHCO_3_, and 5 pyruvate (pH 7.3, 310 mOsm) and saturated with 95% O_2_/5% CO_2_. Brains were mounted on a vibrating slicer (Leica) submerged in ice‐cold protective solution. After slicing, 300 μm hippocampal coronal slices were first incubated at 32°C for 30 minutes in regular aCSF (pH 7.4, 310 mOsm) containing (in mM) 26 NaHCO_3_, 2.5 KCl, 126 NaCl, 20 D‐glucose, 1 sodium pyruvate, 1.25 NaH_2_PO_4_, 2 CaCl_2_, and 1 MgCl_2_. The hippocampal slices were then allowed to maintain at room temperature for 0.5–5 hours. Hippocampal slices were transferred to a recording chamber and bathed in a regular aCSF, which saturated with 5% CO_2_ and 95% O_2_ and perfused at 3 mL/min (Defert & Boland, 2017; Rafael et al., 2015 ). Whole‐cell patch‐clamp recordings were performed in the current‐clamp mode using 700B amplifier (Molecular Devices). Recordings pipettes (5–6 MΩ) were filled with a solution containing substances in mmol/L: 125 K‐gluconate,10 HEPES, 2 MgCl_2_, 4 ATP‐Na_2_, 0.4 Na+‐GTP, 10 phosphocreatine disodium salt, 0.5 EGTA, 10 KCl (pH 7.3–7.4, 280–290 mOsm). Experiments were performed at physiological temperature 32°C. Data was sampled at 10 kHz and filtered (low pass) at 6 kHz and, then analyzed offline using pClamp 10.6 software (Molecular Devices). For all recordings performed with K‐gluconate in the pipettes, potentials were corrected for a junction potential of −10 mV.

### 2.10 Statistics

Results were presented as mean ± standard deviation (SD). Differences among experimental groups were compared by homogeneity test of variance and then one‐way analysis of variance (ANOVA) using SPSS software followed by Student–Newman–Keuls test for post hoc comparisons (Version 19.0, SPSS Statistics.lnk, NY, USA). Differences with *p* < .05 were considered statistically significant. All graphs were made with Graphpad (Prism 7.0, Graphpad Software) and photoshop (Photoshop CS6, Adobe Systems).

## 3 RESULTS

### 3.1 KA significantly activated the RhoA/Rho kinase pathway in Neuro‐2a cells

**FIGURE 1:**
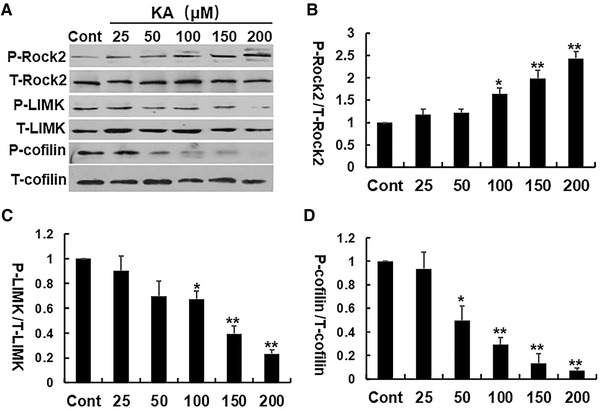
KA significantly activated RhoA/Rho kinase pathway in a dose‐dependent fashion. (A) Representative Western blots of P‐ROCK2, T‐ROCK2, P‐LIMK, T‐LIMK, P‐cofilin, and T‐cofilin expression. (B–D) The relative expression of phosphorylated proteins was calculated and normalized to the control. *p < .05, **p < .01 by one‐way ANOVA compared to control group (*n* = 5)

Our previous research demonstrated that KA seizures induced a rapid activation of cofilin and corresponding depolymerization of actin filaments in dendrites in mice (Zeng et al., 2007). To determine the appropriate dosage and timing of KA treatment which could cause activation of the RhoA/Rho kinase pathway, we carried out a dose‐ and time‐dependent Western blot analysis in Neuro‐2a cells. In this series of experiments, we examined the phosphorylation level of RhoA/Rho kinase pathway components in Neuro‐2a cells after treated with 0, 25, 50, 100, 150, and 200 μmol/L KA. The immunoblot analysis revealed a significant increase in phosphorylated ROCK2 starting from 100 μmol/L, and displayed a dose‐dependent increase (Figures 1A, B) after serum deprivation for 1 hour. The phosphorylated LIMK and Cofilin significant decreased after treatment with 150 and 100 μmol/L KA, respectively. (Figures 1A, C, D). Thus, we selected KA at a dose of 200 μmol/L for the following experiments.

**FIGURE 2:**
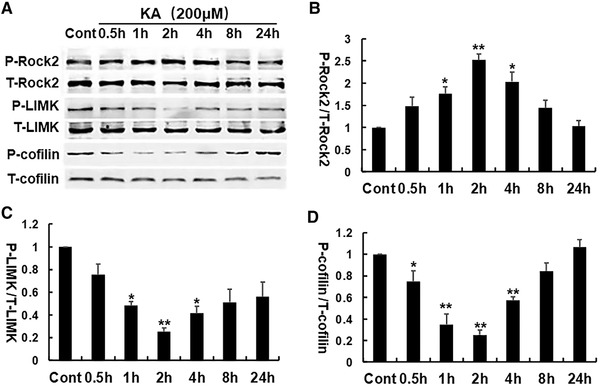
The time course of KA‐induced activation of the RhoA/Rho kinase pathway. (A) Representative Western blots of P‐ROCK2 T‐ROCK2, P‐LIMK, T‐LIMK, P‐cofilin, and T‐cofilin expression. (B–D) The relative expression of phosphorylated proteins was calculated for each condition and normalized to the control. *p < .05, **p < .01 by one‐way ANOVA compared to control group (*n* = 5)

To determine the time course of KA on the RhoA/Rho kinase pathway, the Neuro‐2a cells were treated with 200 μmol/L KA for 0, 0.5, 1, 2, 4, 8, and 24 hours and collected for western‐blot analysis. The phosphorylated ROCK2 protein started to increase at 0.5 hour and peaked at 2 hours, then gradually turned to the baseline (Figures 2A, B). Simultaneously, the phosphorylation levels of LIMK1 and cofilin proteins in Neuro‐2a cells started to decrease at 0.5 hour and peaked at 1–2 hours, and then turned to baseline gradually. These data further indicated that KA significantly changed the phosphorylation levels of RhoA/Rho kinase pathway (Figures 2A–D).

### 3.2 Pretreatment of fasudil effectively reversed KA‐induced change of the RhoA/Rho kinase pathway and alleviated KA‐induced actin depolymerization

**FIGURE 3:**
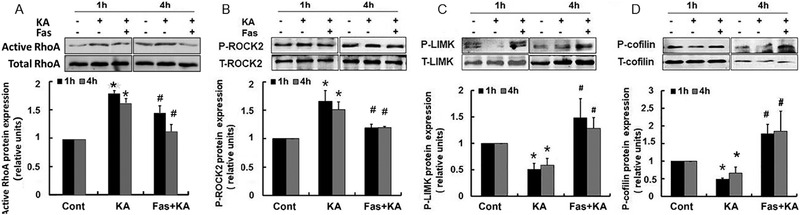
Pretreatment of fasudil effectively rescued KA‐induced activation of RhoA/Rho kinase pathway. (A–D) Top: Representative western blots of phosphorylation and total protein of RhoA, ROCK2, LIMK, and cofilin, respectively. Bottom: Quantification of phosphorylation ratio of each protein. *p < .05 by one‐way ANOVA compared to control group (*n* = 5). #*p* < .05 by one‐way ANOVA compared to KA‐treated group (*n* = 5)

The effect of fasudil on RhoA/Rho kinase pathway was investigated. As shown in Figure 3, pretreatment with fasudil significantly inhibited the activation of RhoA (Figure 3A) and phosphorylated ROCK2 (Figure 3B), and promoted phosphorylated LIMK and cofilin almost both 1 or 4 hours after KA treatment (Figures 3C, D). These results demonstrated that fasudil effectively rescued the KA‐induced change of the RhoA/Rho kinase pathway.

**FIGURE 4:**
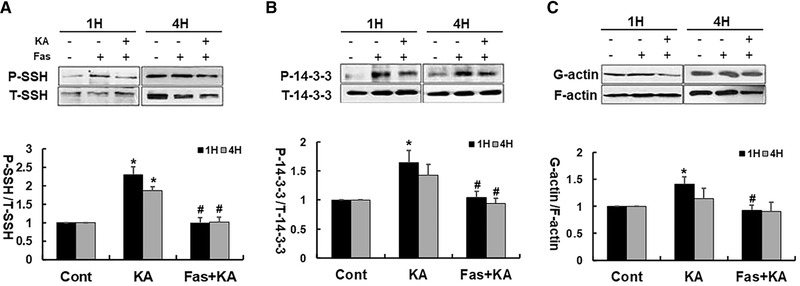
Fasudil significantly downregulated the KA‐induced increase of P‐SSH and P‐14‐3‐3 protein in Neuro‐2a cells. (A) Top: Representative western blots of P‐SSH1 and T‐SSH1 protein. Bottom: Quantification of P‐SSH1 protein expression. (B) Top: Representative western blots of P‐14‐3‐3 and T‐SSH1 protein. Bottom: Quantification of P‐14‐3‐3 protein expression. (C) Top: Representative western blot for actin (G‐actin and F‐actin). Bottom: Summarized data for all experiments show that KA caused markedly decrease of filamentous actin 1 hour after KA treatment, and fasudil alleviated KA‐induced actin depolymerization. *p < .05 by one‐way ANOVA compared to control group (*n* = 5). #*p* < .05 by one‐way ANOVA compared to KA‐treated group (*n* = 5)

It has been showed by several groups that protein slingshot phosphatase isoform 1 (P‐SSH1) could dephosphorylate P‐cofilin and reactivate cofilin (Dhruba & Peng, 2016; Govek, 2005 ). Thus, we investigated whether KA treatment had an effect on the expression of phosphorylated SSH1 and its upstream protein P‐14‐3‐3. It was found that the KA treatment significantly increased the expression of P‐SSH1 and P‐14‐3‐3 at both 1 and 4 hours (Figures 4A, B). By contrast, pretreatment with fasudil significantly reversed the KA‐induced increase of phosphorylated P‐SSH1 and P‐14‐3‐3 in Neuro‐2a cells (Figures 4A, B).

In addition, KA caused a marked decrease of filamentous actin at 1 hour after treatment, and fasudil blocked the actin depolymerization. The actin depolymerization recovered partially 4 hours after KA treatment but the effect of fasudil still persisted (Figure 4C).

### 3.3 Fasudil pretreatment reversed KA‐induced neurite outgrowth inhibition in Neuro‐2a cells

**FIGURE 5:**
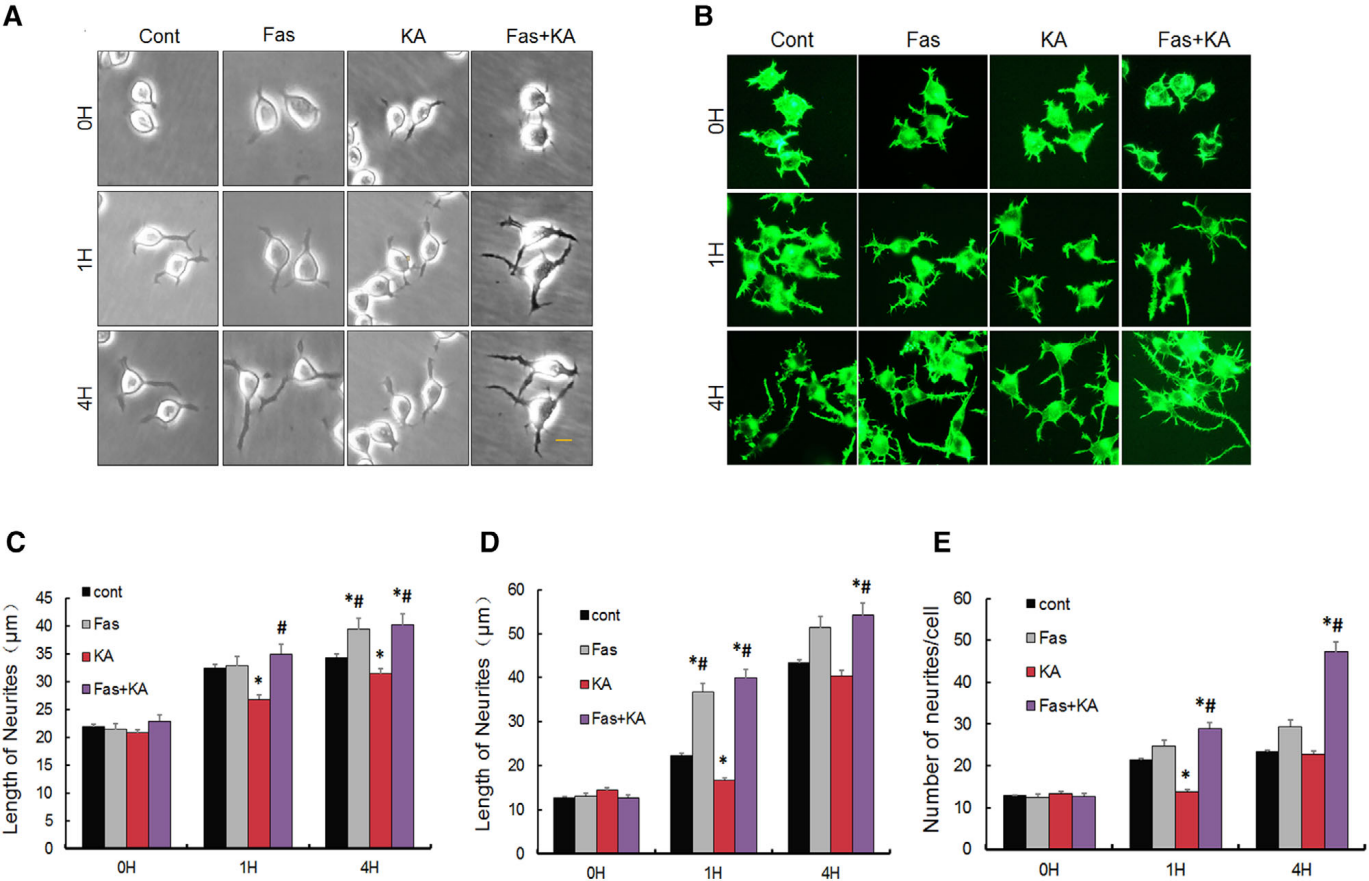
Fasudil reversed KA‐induced inhibition of neurite outgrowth and spines in Neuro‐2a cells. (A, B) Representative intrinsic optical and rhodamine‐phalloidin immunofluorescent images of Neuro‐2a cells, respectively. Scale bar = 20 μm. (C, D) Quantitative analysis of the length of outgrowth in Neuro‐2a cells under the observation of intrinsic optical microscope and rhodamine‐phalloidin immunofluorescence, respectively. (E) Quantitative analysis of the number of spines in Neuro‐2a cells. *p < .05 by one‐way ANOVA compared to control group (*n* = 100). #*p* < .05 by one‐way ANOVA compared to KA‐treated group (*n* = 100)

Neuro‐2a cells incubated without serum demonstrated neuronal morphologies and the increased lengths of neurites proportionally to the duration of incubation. Neuro‐2a cells tended to clump during extended culture, and thus the differentiation period should be limited to 48−72 hours (Rafael et al., 2015). Using different interface contrast microscopy for observing the neurite outgrowth in the same field of cells, we found that the length of neurites was significantly shorter in the KA‐treated group than the control group at 1 hour after treatment (Figures 5A, C), whereas the lengths of neurites revealed no significant difference from the control at 4 hours, indicating that the inhibition of KA on neurite outgrowth recovered partly within 4 hours (Figures 5A, C). Compared with the KA‐treated group, neurite extensions were longer in the presence of fasudil (Figures 5A, C).

The rhodamine‐phalloidin immunofluorescence result showed the consistent results (Figures 5B, D). At 1 hour after KA treatment, the length of cell neurites was significantly decreased, compared with control group, and fasudil reversed KA‐induced inhibition (Figure 5D). The lengths of cell neurites in the KA‐treated cells did not differ significantly from that of the control cells at 4 hours after treatment, and neurite extensions were longer in the presence of fasudil when compared with the KA‐treated group and control group at 4 hours (Figure 5D). In addition, we also found that the number of spines was significantly decreased in the KA‐treated cells at the following 1 and 4 hours, and fasudil reversed KA‐induced inhibition on the number of spines (Figures 5B, E). Compared with the KA‐treated group and control group, Neuro‐2a cells showed more spines when treated with fasudil for 1 and 4 hours (Figure 5E). More interestingly, fasudil treated alone also induced the extension of neurite and spine growth. Combined, these results indicated that there were more and longer neurite extensions in the cells treated with fasudil.

### 3.4 Fasudil pretreatment reversed KA‐induced neurite outgrowth inhibition in primary hippocampal cells

**FIGURE 6:**
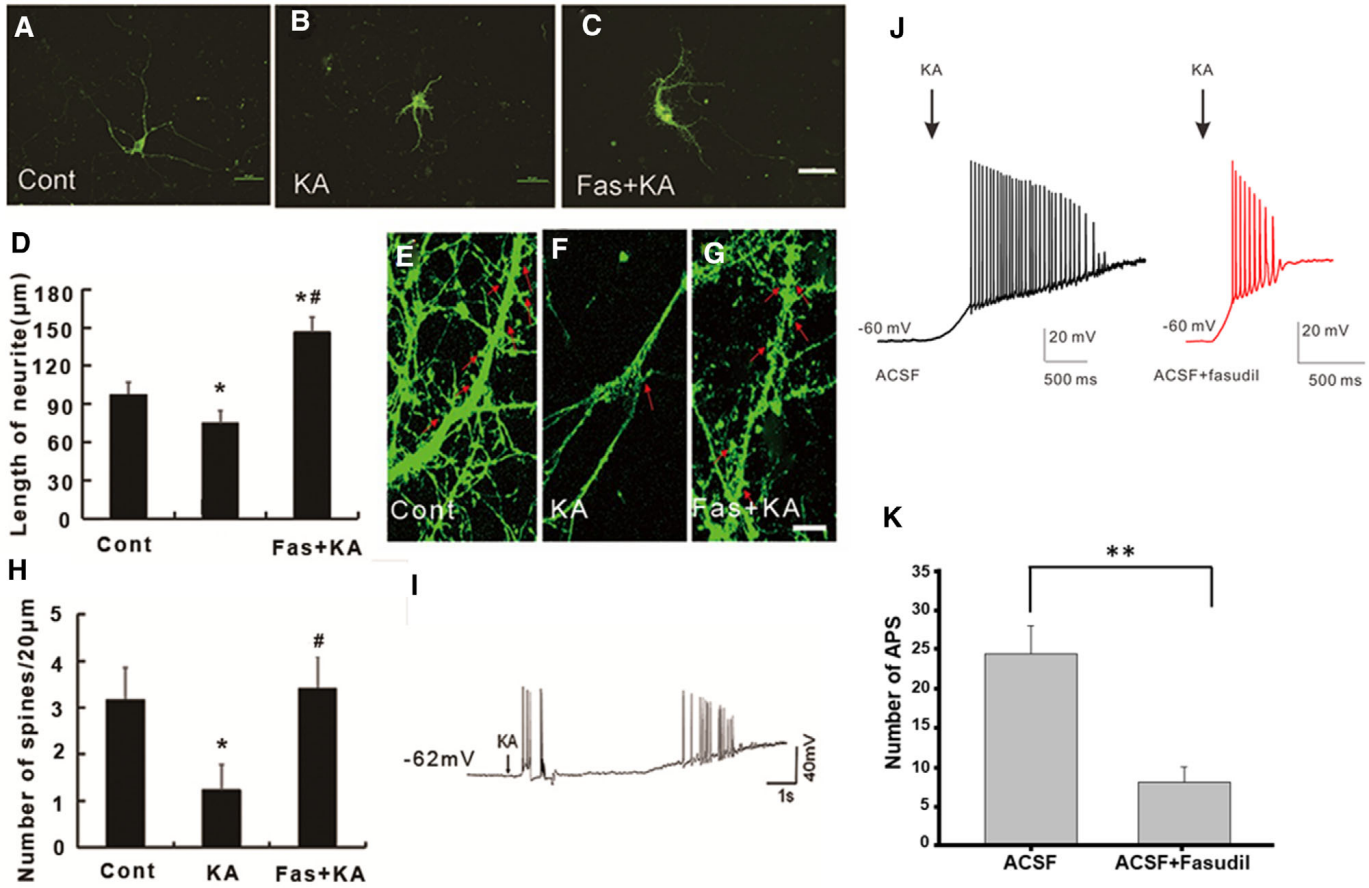
Fasudil pretreatment rescued KA‐induced neurite outgrowth inhibition and spine loss in primary cultured hippocampal neurons. (A–C) The representative rhodamine‐phalloidin image of primary cultured hippocampal neurons at DIV 7 days. Scale bar = 50 μm. (D) Quantification of the neurite length in primary cultured hippocampal neurons *p < .05 by one‐way ANOVA compared to control group (*n* = 20). #*p* < .05 by one‐way ANOVA compared to KA‐treated group (*n* = 20). (E–G) Presentative rhodamine‐phalloidin images of spines on primary cultured hippocampal neurons at DIV 14 days. Scale bar = 10 μm. (H) Quantification of the number of spines in primary cultured hippocampal neurons in different conditions. (I) Presentative trace showed the seizure‐like activity on primary cultured hippocampal neurons evoked by KA application. (J) Presentative trace of the AP with or without fasudil before KA application on hippocampal slices. (H) Quantification of numbers of AP with or without fasudil before KA application on hippocampal slices. *p < .05 by one‐way ANOVA compared to control group (*n* = 6). # *p* < .05 by one‐way ANOVA compared to KA‐treated group (*n* = 6)

To further explore how fasudil affected neurite outgrowth in the primary hippocampal cells, we treated the cells with 200 μmol/L KA for 2 hours at DIV 7 and 14 and observed its effect on neurite outgrowth and dendrite spines. In the neurons of DIV 7, we found that the length of neurites was significantly shorter in the KA‐treated group than the control group, and fasudil reversed KA‐induced neurite outgrowth inhibition (Figures 6A–D). Consistently, in the neurons of DIV 14, the number of spines in primary hippocampal cells was significantly decreased in KA‐treated groups and fasudil reversed KA‐induced inhibition of neurite outgrowth and spine numbers (Figures 6E–H). These results further demonstrated that inhibition of RhoA/Rho kinase signaling pathway protected against KA‐induced neurite injury.

To explore whether KA induced seizure‐like electrical activity in the cultured hippocampal cells, we performed whole‐cell patch‐clamp recording from the cultured hippoampal cells. Indeed, it was found seizure‐like electrical activity from patched neurons after adding 200 μM KA (Figure 6I). Next, we explored whether fasudil had antiseizure like activity in more physiological condition using hippocampal slice. We applied fasudil before KA and found that KA induced less action potentials when in the presence of fasudil compared to the control group (Figures 6J, H).

**FIGURE 7:**
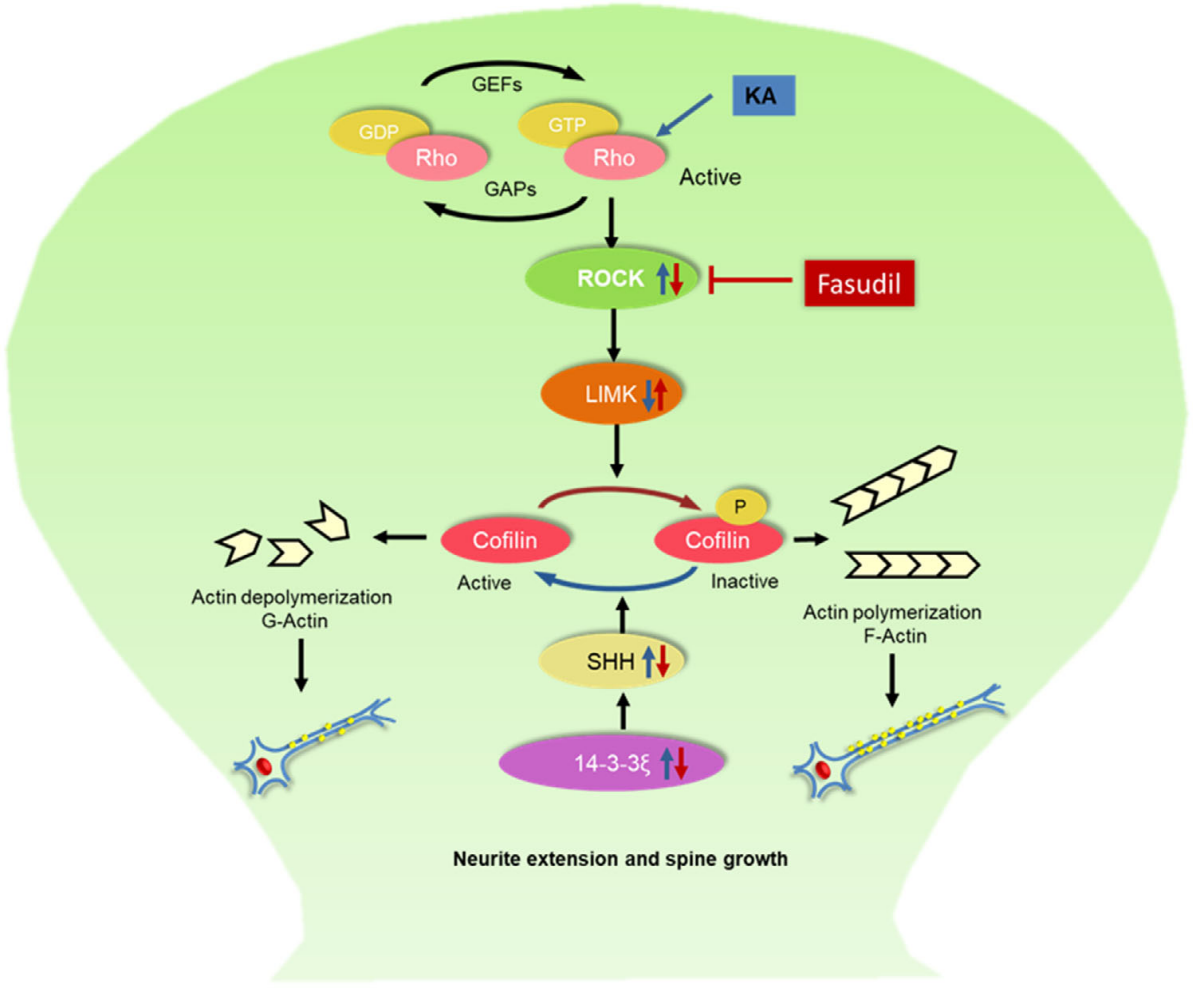
Schematic mechanism of RhoA‐Rho kinase siganling pathway in neurite extension and spine growth *in vitro*

Combined, our results indicate that KA administration induce the activation of RhoA, which subsequently cause the increase of ROCK and decrease of p‐LimK and p‐Cofilin. On the other hand, KA administration induces the increase of 14‐3‐3 and SSH. Both accelerate cofilin dephosphorylation and actin polymerization, leading to inhibition of neurite extension and spine growth. Rock inhibitor fasudil reverses KA‐induced change, keeping the normal neurite extension and spine growth (Figure 7).

## 4 DISCUSSION

In the present study, we found that (1) KA treatment induced actin depolymerization by both activation of the RhoA/Rho kinase pathway and inhibition of phosphatases SSH and 14‐3‐3 and (2) Fasudil pretreatment alleviated KA‐induced actin depolymerization, and subsequently affected neurite outgrowth and dendritic spines in Neuro2A cells and primary cultured neurons. This is the first report, to our knowledge, to show the effect of KA on actin depolymerization in a complete way and that fasudil has protective effect on KA seizure‐induced dendritic spine loss combined with using electrophysiology. Previous studies suggested that KA‐induced high‐stage seizures caused activation of the actin‐depolymerizing factor cofilin and the following actin depolymerization, which was associated with morphologic changes in dendrites (Zeng et al., 2007). Cofilin modulation was a probable mechanism for mediating this seizure‐induced actin depolymerization and dendritic injury. In addition, several studies revealed the antiepileptic effect of Rho kinase inhibitors (Defert & Boland, 2017; Dhruba & Peng, 2016 ). The RhoA/Rho kinase pathway is one of the classic cellular pathways regulating cofilin (Liu et al., 2018). Both dendritic spines and synapses are regulated by the actin cytoskeleton mediated by the Rho GTPases and LIMK1/cofilin (Bernard, 2007; Carcak et al., 2018; Govek, 2005; Rust, 2015). Thus, it is reasonable to assume that RhoA/Rho kinase pathway plays crucial roles in seizure‐induced neurite injury (Jeon et al., 2013). In the present study, we demonstrate that KA can cause immediate changes in RhoA/Rho kinase pathway on the time scale of minutes, which leads to actin decrease in filamentous, neurite outgrowth inhibition and spine loss in Neuro‐2a cells or cultured hippocampal neurons. Furthermore, inhibition of ROCK2 signaling pathway by fasudil reverses KA‐induced injury on neurite outgrowth

The role of the RhoA/Rho kinase pathway in neurite outgrowth has been addressed in numerous studies, but the specific effects on this pathway after seizures remain little known (Lars, 2011). The RhoA/Rho kinase pathway consists of a cohort of kinases and phosphatases maintaining the balance of F‐actin dynamics (Gopalakrishnan et al., 2008; Meng et al., 2002). Cofilin is inactivated by P‐LIMK‐mediated phosphorylation at serine 3 (Ser3) and dephosphorylated by SSH1, which reactivates cofilin‐1 (Bernard, 2007; Mitsuharu et al., 2003; Soosairajah et al., 2005). LIMK and SSH1 show the highest substrate specificity compared to other kinases and phosphatases that affect cofilin‐1 activity (Mitsuharu et al., 2003; Soosairajah et al., 2005; Zafar et al., 2017). Moreover, SSH1 is also regulated by 14‐3‐3 protein (Mitsuharu et al., 2003). ROCK2 (brain type), an upstream kinase, can regulate LIMK and then inactivate cofilin by phosphorylation (Koch et al., 2014; Shi et al., 2013). Our results and others do not show whether RohA/Rho kinase phosphorylate SSH1 and 14‐3‐3. This study analyzed the phosphorylation levels of ROCK2, LIMK, cofilin, SSH1, and 14‐3‐3 after the cells were treated with KA for different durations. LIMK and cofilin markedly dephophorylated within 2 hours and then the phosphorylation level gradually returned to the baseline levels. However, ROCK2 dramatically phosphorylated within 0.5 hour and peaked at the time point 1 hour. Interestingly, we found that the phosphorylation level of ROCK2 peaked at 1 hour and the dephosphorylation levels of LIMK and cofilin reached to the peak also at 1 hour after KA treatment. In consistent with a previous result (Singh et al., 2017), our data showed that ROCK2 could regulate LIMK and T‐LIMK further inactivated T‐cofilin. In addition, we also analyzed the dose‐dependent effects of KA on the RhoA/Rho kinase pathway. The results showed that with the increase of the dosage, the phosphorylation (ROCK2)/dephosphorylation (LIMK and Cofilin) level of RhoA/Rho kinase pathway increased/decreased 1 hour after KA treatment. KA is a structural analogue of the aminoglutaminic acid that activates receptors for glutamate and can work as an excitatory neurotransmitter in the central nervous system (Koch et al., 2014). Thus, it can be used as a neurodegenerative agent and to establish epilepsy and Alzheimer's disease modeling (Lévesque & Avoli, 2013; Park et al., 2012 ). This study revealed that the length of the KA‐treated Neuro‐2a cells neurite outgrowth was dramatically shorter than the controls. Thus, we demonstrated that the amount of p‐LIMK decreased after KA treatment through the regulation of RhoA/Rho kinase pathway. This subsequently caused the activation of cofilin and further depolymerization of F‐actin, which was associated with dramatic neurite outgrowth inhibition. It was speculated that RhoA/Rho kinase pathway may be one of the key pathways meditating neurodegeneration in epilepsy.

The inhibitors of ROCK2, such as fasudil and Y‐27632, have been reported with an antiepileptic effect (Xu et al., 2014). However, the specific intracellular signaling and mechanistic elements involved are still unclear. In this research, we applied fasudil to Neuro‐2a cells before KA treatment and analyzed the phosphorylation level of key proteins in RhoA/Rho kinase pathway. The results showed that fasudil pretreatment could significantly reverse the decrease of phosphorylation level of LIMK and cofilin proteins in RhoA/Rho kinase pathway induced by KA. According to the study, we proposed that the inhibitors of ROCK2 caused the decrease in the phosphorylation level of ROCK2, which led to an increase in the phosphorylation of LIMK and cofilin protein through the RhoA/Rho kinase pathway.

The increase of phosphorylation level of SSH1 and 14‐3‐3 after KA treatment also returned to baseline because of an increase in fasudil‐induced phosphorylation of cofilin protein which can also maintain the polymerization of actin and the normal structure of neurite of Neuro‐2a cells. Thus, in Neuro‐2a cells, the ROCK2 inhibitors can reverse KA's effects during RhoA/Rho kinase signaling pathway by three ways: first, increasing the phosphorylation level; second, protecting actin from depolymerization; and third, increasing the neurite outgrowth.

The above results indicate that the RhoA/Rho kinase pathway regulated the neurite growth inhibition after KA treatment and inhibitors of RhoA/Rho kinase pathway are effective in protecting against KA‐induced neurite growth inhibition. These findings shed light on the mechanisms of modulation of actin‐based neurite growth and provide the potential drug targets for treating neurodegenerative diseases. Most of all, this study provides exciting preclinical data that support the initiation of clinical trials using fasudil for treating comorbidity of epilepsy after additional animal and clinical studies since fasudil is a FDA‐approved drug. Nevertheless, a potential limitation of these experiments was the use of Neuro‐2a cells and primary hippocampal cells in vitro. Future studies will examine these mechanisms further in in vivo epilepsy models.

## CONFLICT OF INTEREST

All authors claim that there are no conflicts of interest.

### PEER REVIEW

The peer review history for this article is available at https://publons.com/publon/10.1002/brb3.2266.
